# Mutations matter: An observational study of the prognostic and predictive value of KRAS mutations in metastatic colorectal cancer

**DOI:** 10.3389/fonc.2022.1055019

**Published:** 2022-11-29

**Authors:** Daniele Lavacchi, Sara Fancelli, Giandomenico Roviello, Francesca Castiglione, Enrico Caliman, Gemma Rossi, Jacopo Venturini, Elisa Pellegrini, Marco Brugia, Agnese Vannini, Caterina Bartoli, Fabio Cianchi, Serena Pillozzi, Lorenzo Antonuzzo

**Affiliations:** ^1^ Clinical Oncology Unit, Careggi University Hospital, Florence, Italy; ^2^ Department of Experimental and Clinical Medicine, University of Florence, Florence, Italy; ^3^ Department of Health Science, University of Florence, Florence, Italy; ^4^ Pathologic Histology and Molecular diagnostic Unit, Careggi University Hospital, Florence, Italy; ^5^ Medical Oncology Unit, Careggi University Hospital, Florence, Italy

**Keywords:** KRAS, colorectal cancer, G12C, G12V, Exon 4, bevacizumab, codon 146, codon 117

## Abstract

**Background:**

About half of metastatic colorectal cancers (CRCs) harbor Rat Sarcoma (RAS) activating mutations as oncogenic driver, but the prognostic role of RAS mutations is not fully elucidated. Interestingly, specific hotspot mutations have been identified as potential candidates for novel targeted therapies in several malignancies as per G12C. This study aims at evaluating the association between KRAS hotspot mutations and patient characteristics, prognosis and response to antiangiogenic drugs.

**Methods:**

Data from RAS-mutated CRC patients referred to Careggi University Hospital, between January 2017 and April 2022 were retrospectively and prospectively collected. Tumor samples were assessed for RAS mutation status using MALDI-TOF Mass Spectrometry, Myriapod NGS-56G Onco Panel, or Myriapod NGS Cancer Panel DNA.

**Results:**

Among 1047 patients with available RAS mutational status, 183 KRAS-mutated patients with advanced CRC had adequate data for clinicopathological and survival analysis. KRAS mutations occurred at codon 12 in 67.2% of cases, codon 13 in 23.5%, codon 61 in 2.2%, and other codons in 8.2%. G12C mutation was identified in 7.1% of patients and exon 4 mutations in 7.1%. KRAS G12D mutation, as compared to other mutations, was significantly associated with liver metastases (1-sided p=0.005) and male sex (1-sided p=0.039), KRAS G12C mutation with peritoneal metastases (1-sided p=0.035), KRAS G12V mutation with female sex (1-sided p=0.025) and no surgery for primary tumor (1-sided p=0.005). No associations were observed between specific KRAS variants and age, ECOG PS, site of primary tumor, pattern of recurrence for resected patients, and lung, distant lymph node, bone, or brain metastases.

Overall survival (OS) was significantly longer in patients with KRAS exon 4 mutations than in those with other KRAS mutations (mOS 43.6 months vs 20.6 months; HR 0.45 [0.21-0.99], p=0.04). No difference in survival was observed for mutations at codon 12/13/61 (p=0.1). Treatment with bevacizumab (BV) increased significatively mPFS (p=0.036) and mOS (p=0.019) of the entire population with a substantial benefit in mOS for G12V mutation (p=0.031).

**Conclusions:**

Patterns of presentation and prognosis among patients with specific RAS hotspot mutations deserve to be extensively studied in large datasets, with a specific attention to the uncommon isoforms and the role of anti-angiogenic drugs.

## Introduction

Colorectal cancer (CRC) is the third most common cause of cancer-related death worldwide with nearly one million deaths per year (1). Approximately half of metastatic CRCs harbor KRAS (Kirsten Rat Sarcoma viral oncogene homologue) activating mutations resulting in the disruption of the homeostatic balance between the GTP-bound active form and the GDP-bound inactive form. The persistence of the RAS active-form, completely disjointed from upstream RTKs influences, causes the overactivation of several downstream pathways involved mainly in cell proliferation and migration processes (2, 3). Thus, drugs targeting RTKs as anti-epidermal growth factor receptor (EGFR) monoclonal antibodies (moAbs) are ineffective. Codons 12 and 13 on exon 2 and codon 61 on exon 3 are the most frequently reported KRAS mutation sites, instead of codons 117 and 146 on exon 4 and mutations on the other RAS family members HRAS and NRAS that are very rare (4–7).

The comprehension of the clinical impact of KRAS mutations in metastatic CRC patients started with the identification of exon 2 mutations as negative predictors of response to anti-EGFR moAbs as per cetuximab and panitumumab (8, 9). Then, an expanded assessment of KRAS mutational status to codons 59, 61, 117, and 146 broadened to NRAS and HRAS mutations, led to restricted prescription of the anti-EGFR moAbs to all-RAS wild-type CRC patients (10).

In contrast, the evidence on the prognostic role of KRAS mutations is controversial and when widened to molecular subgroup analyses the results are not distinct (11–14).

In view of the frequency of the mutation and its inherent resistance to available treatments in CRC, much effort has been done into the development of new molecules targeting KRAS mutations.

In the last few years, specific hotspot mutations have been distinguished to be potential candidates for novel targeted therapies. Sotorasib, an irreversible KRAS G12C inhibitor, demonstrated a modest clinical activity as single agent in G12C-mutated CRC patients who previously received standard treatments including fluoropyrimidine, oxaliplatin, and irinotecan (15). Ongoing studies are exploring the safety and activity of sotorasib in combination with other therapeutic agents with the aim to overcome anti-EGFR resistance in these patients. The combination of panitumumab and sotorasib is under investigation in a multicenter, randomized, phase III trial for previously treated metastatic CRC patients with KRAS G12C mutation [NCT05198934]. Similarly, the combination of cetuximab and adagrasib, another potent KRAS G12C inhibitor, is under investigation in a randomized phase III trial in patients who progressed after first-line treatment for metastatic CRC [NCT04793958]. Interestingly, preclinical studies are exploring other targeted therapies against different RAS hotspot mutations (e.g. G12V) or different therapeutic approaches (e.g. tri-complex inhibitors, RAS-effector interaction inhibitors, mRNA encoding neo-epitopes for RAS mutations) (16, 17).

Since RAS-mutated metastatic CRC is widely considered to be a heterogeneous group of diseases, this study aims at evaluating the association between specific hotspot mutations and patient characteristics, prognosis and response to antiangiogenic drugs.

## Patients and methods

### Patient population and study design

Data from CRC patients for whom RAS mutational status was analyzed on surgical specimen or biopsy at Careggi University Hospital, between January 2017 and April 2022, were retrospectively and prospectively collected. All available demographic data, medical history, diagnosis, stage, chemotherapy, curative or palliative surgery, pathological results, molecular analysis, clinical outcomes were collected from medical records. Radiological response was assessed according to RECIST, version 1.1 (18).

The two primary endpoints were the evaluation of the prevalence of KRAS mutations according to patient characteristics and the KRAS codon-specific hotspot mutations prognostic role.

Overall survival (OS) was defined as the time from the start of treatment to death from any cause. Progression-free survival (PFS) was defined as the time from the start of treatment to progressive disease, or death from any cause, whichever occurred first.

This retrospective and prospective observational study was approved by the Institutional review board of Azienda Ospedaliero-Universitaria Careggi (Regional Ethical Committee for clinical experimentation of Tuscany – Italy - Area Vasta Centro – 20981_bio). We obtained informed consent from each alive patient enrolled in the study.

### RAS mutation analysis

KRAS assessment was performed by the Histopathology and Molecular Diagnostics Unit of Careggi University Hospital. DNA was extracted from formalin-fixed paraffin-embedded tissue samples using MagCore^®^ Genomic DNA FFPE One-Step Kit on MagCore^®^ Automated Nucleic Acid Extractor HF16Plus according to specific diagnostic protocol. From 2017, RAS mutations were detected using MALDI-TOF Mass Spectrometry with Myriapod^®^ Lung status on MassARRAY^®^. From 2020, KRAS analysis was performed using Myriapod NGS-56G Onco Panel on Ion Torrent Ion S5™ system or Myriapod NGS Cancer Panel DNA on Illumina MiSeq^®^. The analysis of the NGS sequencing results was made using Myriapod NGS Data Analysis Software and the mutations were selected using the online genetic databases Clinvar and COSMIC (a minimum variant allele frequency – VAF - of 5% was applied for variant filtering).

### Statistical analysis

Correlations of demographic, clinical, pathological, molecular factors, survival outcomes and specific KRAS hotspot mutations were analyzed. Statistical comparisons for categorical variables were performed using the χ2 test. Time-to-event endpoints were estimated using the Kaplan-Meier method. Survival distributions for specific subgroups of patients were tested with a log-rank test. A p-value of 0.05 or lower was considered statistically significant. Parameters with a statistically significant log-rank test were considered independent variables and included in the multivariate Cox proportional hazard regression linear model to compare hazard ratio (HR) and 95% confidence interval (95% CI). All analyses were performed using R, version 4.2.1.

## Results

### Demographic and clinical-pathologic characteristics in population according to KRAS mutations

**Figure 1:**
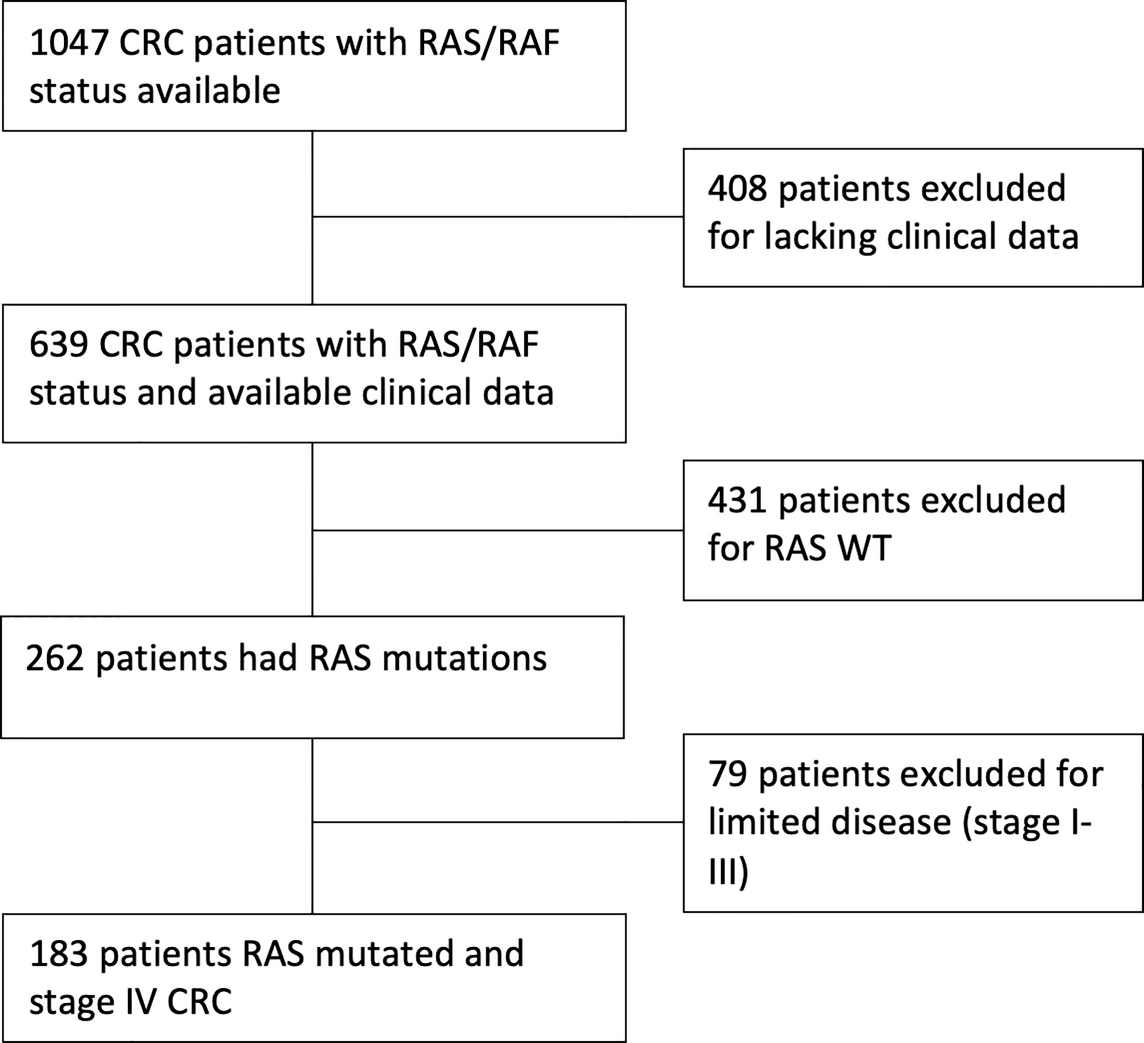
Flow chart of CRC patients with available RAS mutational status within the RAS&Co study.

We collected clinical data from 1047 patients with KRAS assessment available. Of these, 408 were excluded due to incomplete follow up or other missing data. Of the remaining 639 patients, KRAS mutations were identified in 262 cases. Seventy-nine patients had a stage I-III disease and were excluded. Finally, 183 KRAS-mutated patients with CRC stage IV were enrolled for clinicopathological and survival analysis (**Figure 1**).

Population analyzed had a median age of 69 years (range 30-88) and were mainly male (57.4%). Right colon (30.6%) and rectum (39.3%) were the main sites of location of the primary tumor followed by the transverse colon in 7.1% and the left colon in 22.9%. Surgery on primary tumor was performed in 76.0% of cases. The vast majority of patients were stage IV at diagnosis (61.7%), while 38.3% of patients developed metachronous metastases. Seventy-two patients (39.3%) had multi-organ metastases, with liver and lung being the most involved sites. Bone and brain metastases were rare (4.4% and 2.2%, respectively). About half of patients (51.9%) received antiangiogenic agents in the first-line treatment.

**Figure 2:**
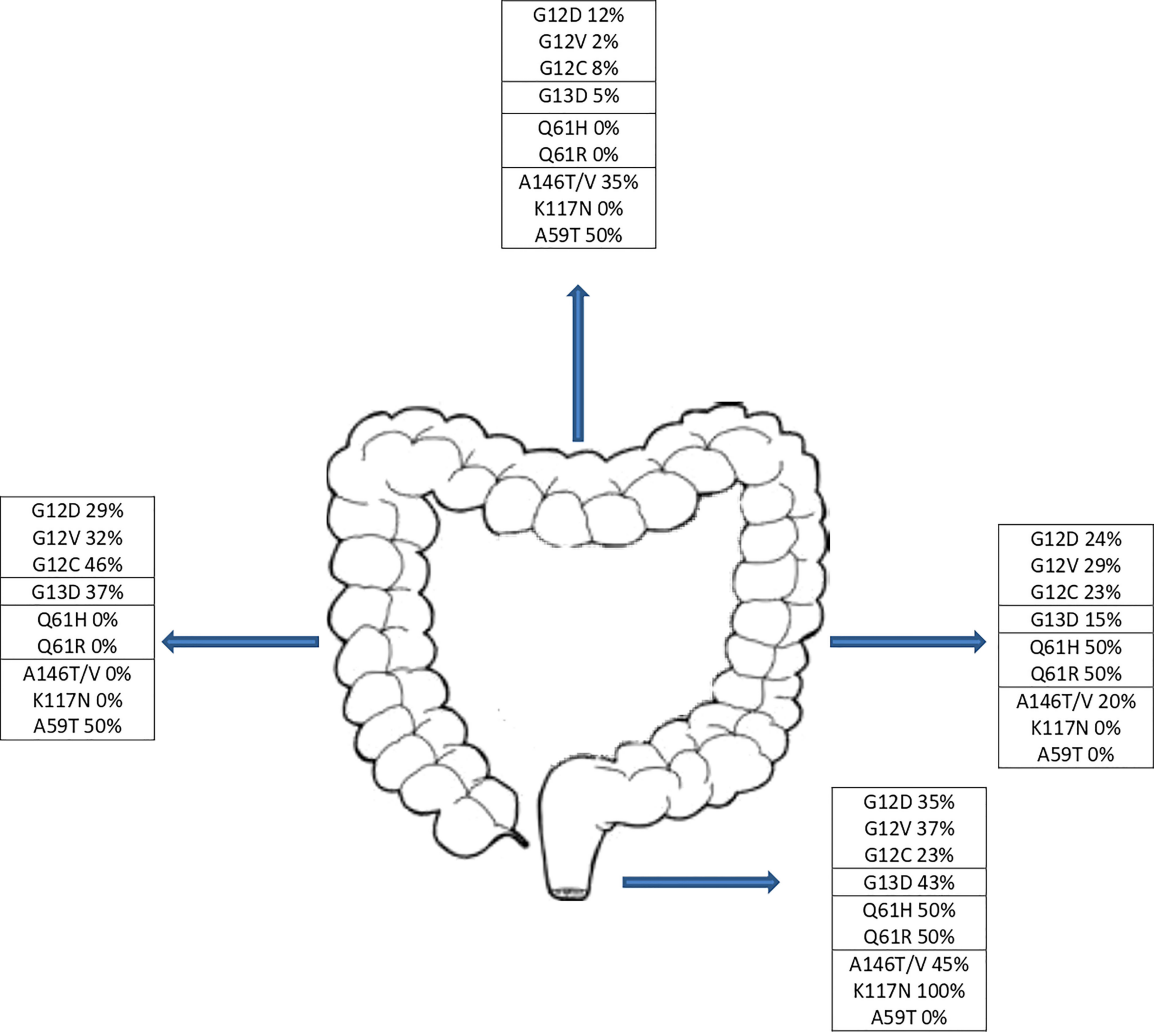
Distribution of KRAS mutations by tumor site.

KRAS mutations occurred at codon 12 in 123 patients (67.2%), codon 13 in 43 patients (23.5%), codon 61 in 4 patients (2.2%) and other codons in 15 patients (8.2%). Two patients had concomitant mutations in two different codons. Across the codons, the distribution of demographic characteristics reflected the features of the global population, although patients with codon 61 mutations showed no primary lesion in right and transverse colon (**Figure 2**) and metastasis distribution exclusively in liver and lung, while brain metastases affected entirely codon 12 mutated patients (G12V n=13, G12D n=1). The baseline demographic and disease characteristics are shown in **Table 1**.

**Table 1:** Baseline demographic and disease characteristics (n=183).

	Overall populationn (%)	Codon 12n (%)	Codon 13n (%)	Codon 61n (%)	Other Codonsn (%)
Sex					
Male	105 (57.4%)	72 (39.3%)	24 (13.1%)	2 (1.1%)	9 (4.9%)
Female	78 (42.6%)	51 (27.9%)	19 (10.4%)	2 (1.1%)	6 (3.3%)
Age					
Median 69y (30-88)					
<70 years	91 (49.7%)	67 (36.6%)	18 (9.8%)	1 (0.5%)	9 (4.9%)
≥70 years	92 (50.3%)	56 (30.6%)	25 (13.7%)	3 (1.6%)	6 (3.3%)
Primary tumor location					
Right colon	56 (30.6%)	36 (19.6%)	16 (8.7%)	0 (0%)	5 (2.7%)
Transverse colon	13 (7.1%)	9 (4.9%)	3 (1.6%)	0 (0%)	1 (0.5%)
Left colon	42 (22.9%)	33 (18.0%)	6 (3.3%)	2 (1.1%)	2 (1.1%)
Rectum	72 (39.3%)	45 (24.5%)	18 (9.8)	2 (1.1%)	7 (3.8%)
Stage at diagnosis					
I	3 (1.6%)	1 (0.5%)	1 (0.5%)	0 (0%)	1(0.5%)
II	28 (15.3%)	18 (9.8%)	6 (3.3%)	2 (1.1%)	3 (1.6%)
III IV	39 (21.3%) 113 (61.7%)	26 (14.2%) 78 (42.6%)	10 (5.5%) 26 (14.2%)	0 (0%) 2 (1.1%)	4 (2.2%) 7 (3.8%)
Site of metastases					
Liver	106 (57.9%)	78 (42.6%)	20 (10.9%)	3 (1.6%)	6 (3.3%)
Lung	72 (39.3%)	48 (26.2%)	16 (8.7%)	2 (1.1%)	7 (3.8%)
Bone	8 (4.4%)	7 (3.8%)	1 (0.5%)	0 (0%)	0 (0%)
Peritoneum	49 (26.8%)	30 (16.4%)	14 (7.6%)	0 (0%)	5 (2.7%)
Distant lymph nodes	35 (19.1%)	27 (14.7%)	6 (3.3%)	0 (0%)	3 (1.6%)
.Brain	4 (2.2%)	4 (2.2%)	0 (0%)	0 (0%)	0 (0%)
Tumor burden					
Single organ	111 (60.6%)	71 (38.8%)	29 (15.8%)	3 (1.6%)	10 (5.4%)
Multi-organ	72 (39.3%)	52 (28.4%)	14 (7.6%)	1 (0.5%)	5 (2.7%)
Surgery on primary tumor					
Yes	139 (76.0%)	93 (50.8%)	30 (16.4%)	3 (1.6%)	15 (8.2%)
No	44 (24.0%)	30 (16.3%)	13 (7.1%)	1 (0.5%)	0 (0%)

Within codon 12, G12D mutation was the most detected affecting 51 patients (27.9%), followed by G12V in 40 patients (21.9%) and G12C in 13 patients (7.1%). The codon 13 resulted most affected on G13D with 40 patients mutated (21.8%) while across mutations in other codons than 12,13 and 61 the exon 4 was the most frequently affected with A146T/V in 11 patients (6.0%) and the K117N in 2 patients (1.1%) mutations mainly detected (**Table 2**).

**Table 2:** KRAS mutations (patients, n=183*).

	Overall population n (%)
Codon 12	
G12D	51 (27.9%)
G12V	40 (21.9%)
G12C	13 (7.1%)
G12S	10 (5.5%)
G12A	7 (3.8%)
Others	2 (1.1%)
Codon 13	
G13D	40 (21.8%)
G13C	1 (0.5%)
G13V	1 (0.5%)
G13S	1 (0.5%)
Codon 61	
Q61H	2 (1.1%)
Q61R	2 (1.1%)
Other codons	
A146T/V	11 (6.0%)
K117N	2 (1.1%)
A59T	2 (1.1%)

*two patients had two concomitant mutations in KRAS.

The G12D mutation, as compared to other mutations, was significantly associated with liver metastases (75.5% in G12D vs 53.5% in other variants, 1-sided p=0.005) and male sex (68.6% in G12D vs 58.0% in other variants, 1-sided p=0.039), while G12C patients were mainly affected by peritoneal metastases (53.8% in G12C vs 25.4% in other variants, 1-sided p=0.035). The G12V mutation was less frequent in male (42.5% in G12V vs 61.5% in other variants, 1-sided p=0.025) and in patients who received surgery on primary tumor (60.0% in G12V vs 81.7% in other variants, 1-sided p=0.005). Interestingly, all patients with KRAS variants at rare codons received surgery on primary tumor (1-sided p=0.012), while a trend in favor of the lack of surgery on primary tumor was observed in G13D mutation (1-sided p=0.056).

No associations were found between single amino acid substitution on KRAS and other demographic or pathologic disease characteristics as per age, ECOG PS, site of primary tumor, pattern of recurrence for resected patients and sites of metastases as per lung, distant lymph node, bone or brain.

### Survival analysis according to codons and mutations

**Figure 3:**
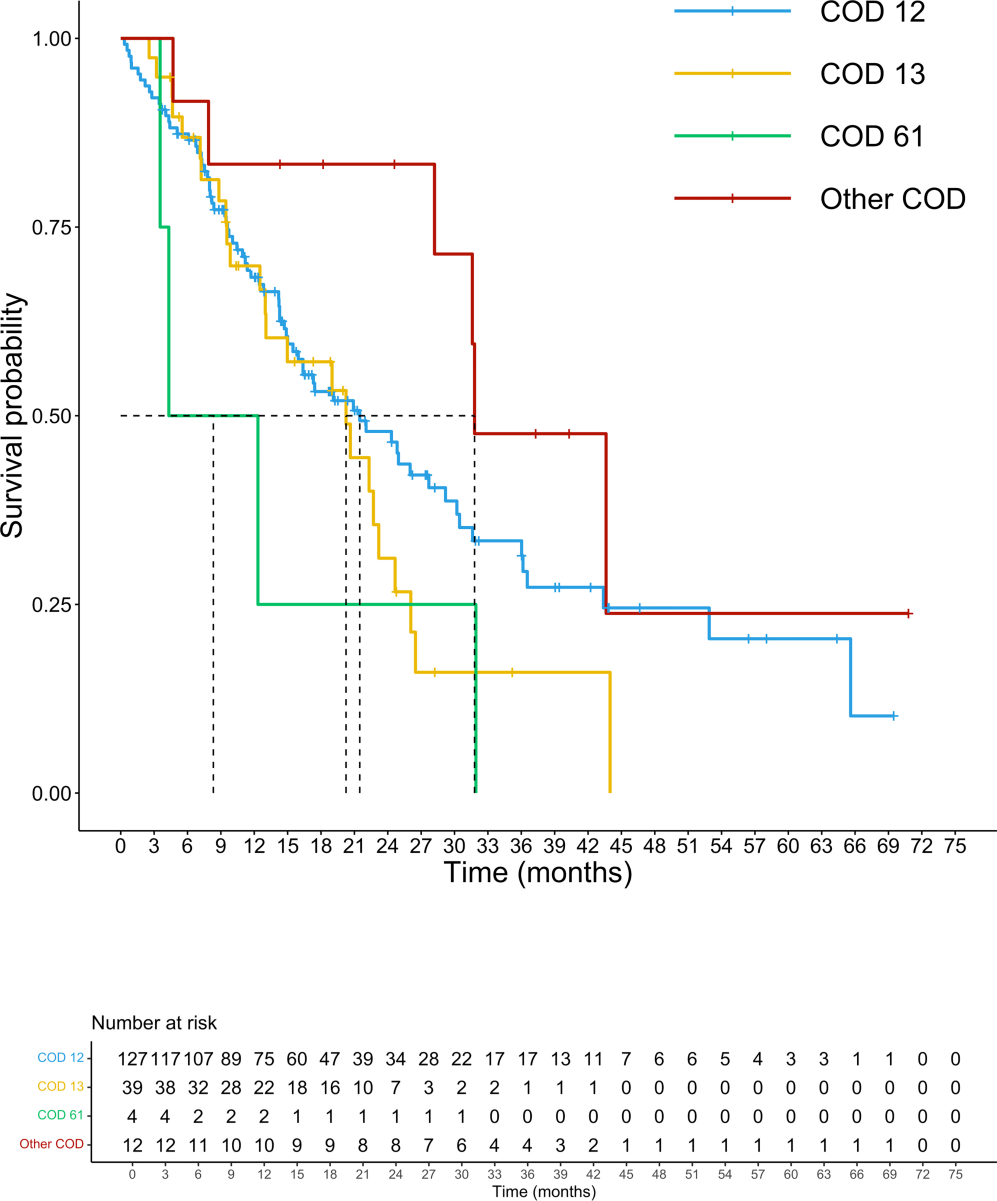
Overall survival according to codons.

**Figure 4:**
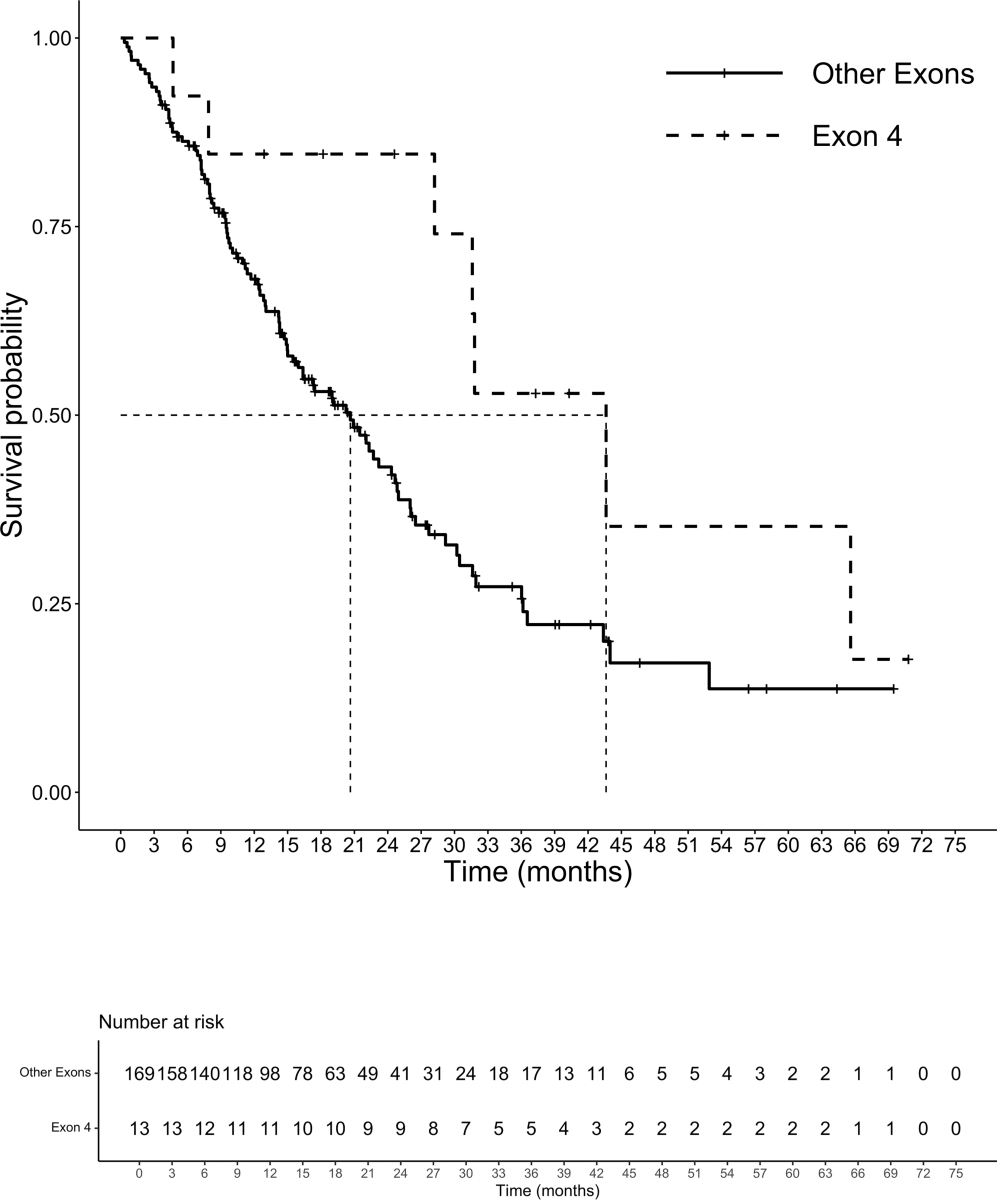
Overall survival exon 4 vs other exons.

**Figure 5:**
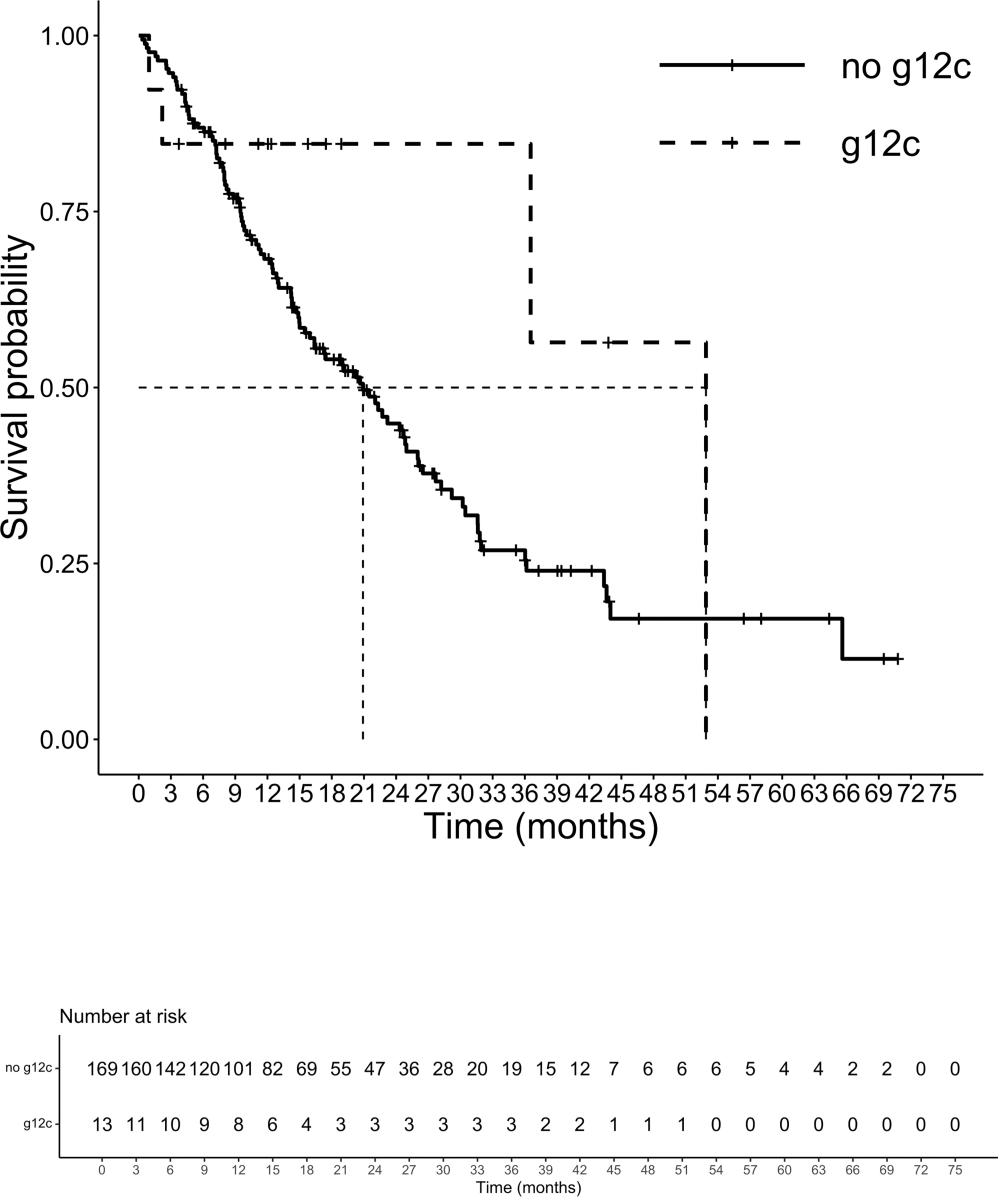
Overall survival G12C vs other mutations.

At a median follow-up of 14.3 months, 105 patients had died. The median (m)OS for the entire cohort was 21.5 months. We analyzed the survival probability of the population according to exons, finding a relevant difference in mPFS for exons 2 and 4 versus exon 3 (ex2: 9.7 months, IC 95% 8.2-10.5; ex4: 10.5 months, IC 95% 4.5-NR; ex3 4.3 months, IC 95% 3.4-NR; p=0.027), benefit not confirmed in mOS (p=0.17). The mOS of codon 12 mutated patients was 21.5 months, (IC 95%: 15.9-27.9) followed by codon 13 patients with a mOS of 20.2 months (12.5-23.2 IC 95%). Patients harboring mutations on codon 61 had the lowest mOS of the entire population analyzed (mOS 4.0 months, IC 95%: 3.5-NR). The group of CRC patients with rare mutation on codons other than 12,13 and 61 registered the highest survival with a mOS of 43.7 months (IC 95%: 7.9-NR) (**Figure 3**). Although the majority of amino acid mutation didn’t show a statistically significant survival performance (p=0.1), K117 and A146 mutations led to a mOS of 43.6 months (IC 95% 28.2-NR) compared to other amino acid substitution mOS of 20.6 months (IC 95%: 5-24.7) (HR 0.45; 95% IC 0.21-0.99; p=0.04) (**Figure 4**). The G12C mOS of 52.9 months (IC 95%: 36.5-NR), compared to 20.6 months (IC 95%: 15-24.3) of the other KRAS hotspot mutations, confirmed the lack of statistically significant difference of the other mainly detected single spot mutations (p=0.1) (**Figure 5**).

### Survival analysis according to anti angiogenic addition to first line chemotherapy

**Figure 6:**
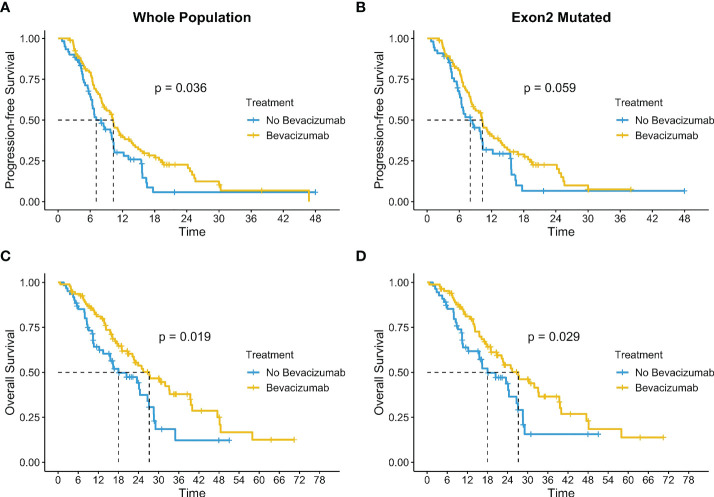
Impact of bevacizumab on the entire population **(A, C)** and according to exons **(B, D)**.

We explored the impact of treatments on the entire population and according to exons and to the most represented hotspot mutations. A total of 155 patients underwent a first-line treatment and of them 63% (n=95) had the antiangiogenic treatment bevacizumab (BV) combined to a standard first-line chemotherapy. The addition of BV induced a significant benefit both in mPFS (10.3 months IC 95%: 8.3-12.4 vs. 7.1 months IC 95% 6.3-10.3; p=0.036) and in mOS (27.2 months IC 95% 22.2-39.6 vs 18.0 months IC 95% 13.5-27.1; p=0.019) as shown in **Figures 6A–C**. BV was added in 60.4% (n=84) of 139 patients with an exon 2 mutation and showed a concrete increase in survival with a mOS of 27.2 months (IC 95% 20.7-39.4) versus 18.0 months (IC 95% 12.2-27.1) of population not treated with BV (p=0.029). mPFS exhibited a trend in favor of the use of BV even without reaching a significance level (10.3 vs 8.0 months, p=0.059) (**Figures 6B–D**).

**Figure 7:**
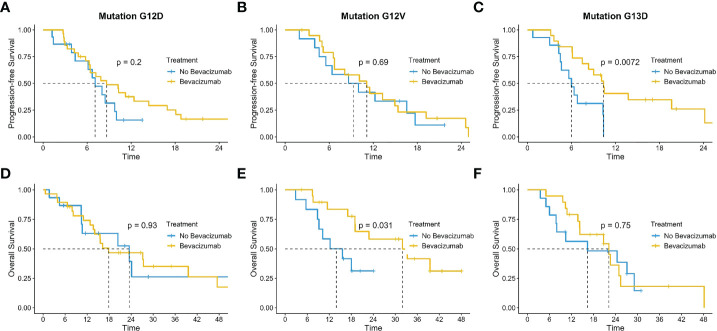
Impact of bevacizumab according to the most frequent mutation detected G12D **(A, D)**, G12V **(B, E)** and G13D **(C, F)**.

We investigated also the impact of BV according to the most frequent mutation detected G12D (n=43), G12V (n=32) and G13D (n=33), identifying a benefit in the addition of BV in G12V mutation with a mOS of 32.0 months (IC 95% 19-NR) versus 14.0 months (9.1-NR) (p=0.031); results not clear in the others (**Figures 7D–F**). The mPFS was exclusively in favor of BV in G13D population (10.4 mo IC 95% 8.3-NR vs 6.0 IC 95% 4.5-NR; p=0.0072) than in the other single hotspot mutations analyzed (**Figures 7A–C**).

## Discussion

RAS is the most commonly mutated gene family in CRCs and has long been considered undruggable and accountable for EGFR moAbs resistance (10, 17, 19). Among the different KRAS variants, G12C has been extensively studied in the last few years as the first druggable mutation despite its low frequency in CRC patients (4, 15). Unfortunately, no targeted therapy is still available for the vast majority of RAS-mutated CRC patients and predictive factors of response to antiangiogenic agents are largely unknown (20–23). Our single-institution study aimed at identifying differences in pattern of presentation and prognosis among metastatic CRC patients according to KRAS hotspot mutations.

In our dataset we did not observe an association between age and specific hotspot mutations, results in line with the case series of Jones et al. and Imamura et al. (11, 12). Differently, a large analysis conducted by Serebriiskii et al. including 6926 KRAS-mutated CRC patients showed a higher incidence of G12 mutations in younger patients and A146, K117, and Q61 mutations in older patients. In contrast, the incidence of G13 mutations was similar across the age groups (4).

In our cohort, G12D mutation was statistically more frequent in males while G12V mutation were mainly detected in females, data in contrast with the study of Serebriiskii et al, where no specific hotspot variant was associated with sex, except for Q61K which was higher in females (4). Right-sided CRC relates with higher incidence of KRAS mutations although it is not possible to identify a prevalence for a specific mutation according to sidedness, data in agreement with the literature (24). However, despite the low number of patients affected by a Q61 mutation (n=4), the totality showed a left-side CRC location as to the best of our knowledge, results not reported in literature (**Figure 2**).

According to the site of metastases at presentation, we observed in G12C a higher incidence of peritoneal metastases and no difference in incidence of lung metastases, as compared to patients with other KRAS mutations. Although the interpretation of these data deserves caution given the G12C small sample size (n=13), a similar trend was reported by Chida et al. In this report, peritoneal metastases were detected in 36% of patients with G12C vs 23% of patients with other KRAS variants (p=0.07) and lung metastases in 40% and 42% (p=0.87), respectively, data confirming our observations (25). In contrast, in a case series of 839 KRAS-mutated patients, of whom 145 harboring G12C variant, Schirripa et al. showed a lower incidence of peritoneal metastases (13% vs 25%, p=0.008) and higher incidence of lung metastases (43% vs 31%, p=0.013) in those with G12C than in those with non-G12C KRAS mutations (26). Similar results were obtained from a large retrospective analysis conducted by Osterlund et al., where peritoneal metastases were reported in 15% of patients with G12C tumor vs 19% of patients with other KRAS mutations (p=0.63) and lung metastases were reported in 39% vs 36%, respectively (p=0.26) (27). Consistent with this, in a large Italian case series the incidence of peritoneal metastases in RAS G12C metastatic CRC patients was 18.9% and lung metastases 41.4% (28).

Intriguingly, despite the low number of patients detected, the 4 patients with CNS metastasis were all G12 mutated, observation not described in literature.

Although it is clear that anti-EGFR moAbs are ineffective in KRAS-mutated patients, the prognostic value of each mutation is still controversial. As reported by Lee et al., within the TCGA and GSE39582 databases, the mutational status of BRAF or KRAS was not associated with OS in CRC patients (29). Several experiences tried to define the role of single KRAS isoforms with conflicting results. A large prospective study (n=440 KRAS-mutated CRC) conducted by Imamura et al. identified KRAS codon 12 mutations as predictive of poor prognosis as compared with wild-type (WT) CRCs. Stratifying by specific mutation, G12V and G12R were associated with higher rates of CRC-specific mortality compared to no mutations (11). Jones et al. collected data from KRAS-mutated patients (n=198) from the Cheshire and Merseyside Cancer Network and compared clinical outcomes with a cohort of WT CRC patients. In multivariate analysis, mutations at codon 12 were significantly associated with reduced survival (HR 1.76, 95% CI 1.27–2.43, p=0.001). In contrast, codon 13 mutations did not have an impact on survival (HR 1.7, 95% CI 0.93–3.46, p=0.06). Among the specific KRAS mutations, G12V and G12C were associated with poor prognosis (HR 1.69, p=0.02 and HR 2.21 p=0.01, respectively) (12). Furthermore, several trials have explored the prognostic effect of specific RAS mutations in stage II-III CRC. In a large cohort of patients within the Intergroup Trial CALGB 89803 (n=508), Ogino et al. did not observe any difference in recurrence or survival between RAS-mutated and WT RAS CRC patients (30). Moreover, Park et al. observed that stage III G12D/V-mutated CRCs had less tumor-infiltrating lymphocytes and shorter recurrence-free survival than WT tumors (31). In contrast, a recent meta-analysis including 9 trials (QUASAR 2, PETACC-8, N0147, CALGB-89803, NSABP-C07, NSABP-C08, PETACC-3, QUASAR, MOSAIC) showed a significant association between KRAS and BRAF mutations and worst survival outcomes (32). The vast majority of retrospective studies involving metastatic patients had WT RAS as the control group. In recent years, several evidence have defined the WT RAS CRC subgroup as a highly heterogeneous population characterized by various gene alterations as fusions (e.g. BRAF; FGFR, NTRK, ALK, RET, ROS1), microsatellite instability (MSI), amplifications (e.g. HER2, MET), and mutations (e.g. PI3KCA, PTEN, AKT) (33–35). Based on these observations, RAS WT can no longer be considered the reference control arm of retrospective studies, therefore considering the relevance of KRAS mutations as an oncogenic driver, we decided to compare specific hotspots mutation of KRAS with the remaining overall KRAS-mutated population.

In our analysis we observed a better PFS for mutation on exons 2 and 4 than exons 3, results not confirmed in OS. When survival was explored according to single canonical mutation, we found no difference in OS. However, a significantly longer OS was observed in patients with KRAS mutations in exon 4 than in those with mutations in other exons. This result is in contradiction with a recently published analysis on the role of mutations on exon 4, especially A146 mutation that seem to have a worse prognosis than G12 isoforms (36). The reason for this divergence can be speculated to be related to biological and clinical factors. Indeed, we know the differences between the various isoforms that dissimilarly affect the RAS signaling pathway by modulating the shift between active and inactive forms *via* GAP (G12 and Q61) or GEF (A146) or both (G13 and K177) (37, 38). Consequently, possessing a non-canonical mutation would seem detrimental. In our population and unlike the previously mentioned study, however, the non-canonical mutations all received surgery on primary tumor, probably affecting survival. Mutations at non-canonical codons are detected in a minority proportion of CRC patients and some of them cause only a moderate alteration of RAS GTPase activity (39). Consistent with our results, some authors reported a more favorable outcome in patients with mutations in exon 4, as compared to those with mutations at codons 12 or 13, but results should be verified in larger case series (40, 41). In addition, in a large database of 1267 cases from two prospective cohort studies including early-stage patients, codon 146 KRAS mutations did not affect prognosis (42). The A146 mutation affects an evolutionarily conserved region that is assumed to interact with the guanine base of GDP that does not impair intrinsic GTPase activity but rather increases the rate of nucleotide exchange resulting in increased activation. In spite of this, the increase in nucleotide exchange activity does not outweigh the decrease in GTPase activity, resulting in increased processing capacity. In CRC cell models have been demonstrated a dependence on MEK/ERK pathway of exon 4 mutations suggesting a possible target in this rare population (40, 43).

We also analyzed the impact of NRAS mutations on prognosis. No difference on OS was observed between patients with KRAS-mutated tumors and those with NRAS-mutated tumors (p=0.89) (**Supplementary**).

The use of antiangiogenics finally showed the benefit of the addition of BV to conventional chemotherapy both in PFS and OS. In the meta-analysis by Petrelli et al, the value of BV addition was evaluated by comparing patients with RAS WT and RAS-mutated tumors showing that mutations are still detrimental with a PFS 11.8 vs 9.42 and OS of 24.5 vs 20.2 months. In our analysis, the use of BV in a mutated population confirmed similar results in mPFS and mOS, showing a significantly increased survival than chemotherapy alone (44). We also sought to identify whether different mutations among those most frequently detected in CRC had different benefits when exposed to BV treatment by observing significant activity in increasing OS in G12V and an increase in PFS in the G13D mutation. As reported in literature among G12 mutations, G12V is the one with the worst survival, and we are able to suggest a possible benefit in survival by adding BV in this specific mutation. G12V has different characteristics than more frequent isoforms such as G12D (45). The slower switch time between active and inactive form that therefore make it even less druggable than the others and the preferential activation of RAF/MEK than PI3K/AKT relate probably with the G12V aggressiveness. Moreover, VEGF receptor-mediated angiogenesis has the RAF/MEK pathway as its regulatory hub, suggesting how different KRAS mutations may be involved more or less deeply in the development of peri-tumor neo vessels (46, 47). Recently an *in mice* experience on arteriovenous malformations, the authors demonstrate that G12V regulates the expression of VEGF-A, VEGFR2 and p-VEGFR2 and stimulates endothelial cells in culture (48). With these assumptions, we can hypothesize that the benefit observed from the use of BV is due to the involvement of G12V in stimulating neo-angiogenesis. The use of BV is closely linked to the absence of risk factors that may facilitate the occurrence of drug-related adverse events, a consideration that may justify the high percentage (37%) of patients not receiving such treatment. We have to emphasize that since this is a retrospective and real-life analysis, a more fragile population due to age and/or clinical conditions and normally excluded from registrational trials, earn a chance for treatment.

Based on the evidence of a high heterogeneity in RAS mutated CRC, the interest in the disease has changed in recent decades. As perspective, the real-time monitoring of RAS-mutated clones by liquid biopsy has highlighted the possibility of an early identification of treatment resistance, hence the need to establish new treatment strategies (49–52). In this regard, we recently presented the results from the OMITERC study, in which we showed the prognostic significance of circulating tumor cells at baseline and cfDNA as a feasible tool for longitudinal monitoring of mutational status and treatment response in a cohort of metastatic RAS-mutated CRC patients (53). Moreover, the increased knowledge in downstream pathways, tumor microenvironment, epigenetics and metabolomics opened to limitless fields of translational research.

In conclusion, despite the limitations related to the observational nature of the study and the natural variability of patients used as a sample in a real-life setting, we can assert that the study confirmed the profound prognostic heterogeneity within the large group of KRAS mutations. We also defined how some mutations, although uncommon and less frequent, need special attention because of their ability to positively (A146) or negatively (G12V and Q61) influence survival. We can also state that although the sample analyzed by definition has inherent sample variability in the type of chemotherapy chosen, the addition of BV in a mutated RAS population in the absence of comorbidity limiting prescription results in a net benefit in PFS and OS. This benefit is maintained in some of the mutations analyzed individually suggesting the need for prospective studies aimed at determining the predictive value of each individual KRAS mutation and identifying any isoforms that may instead benefit from different treatments.

## Data availability statement

The original contributions presented in the study are included in the article/**Supplementary Material**. Further inquiries can be directed to the corresponding author.

## Ethics statement

The studies involving human participants were reviewed and approved by Comitato Etico Regionale for clinical experimentation of Toscana region – Italy - Area Vasta Centro – 20981_bio. The patients/participants provided their written informed consent to participate in this study.

## Author contributions

DL, LA, SP contributed to the design of the study. All authors participated in the collection of clinical and molecular data and data analysis. DL, LA, SP, and SF contributed to the manuscript preparation. All authors contributed to the interpretation of the clinical and molecular data. GiR and SF performed statistical analysis. All authors contributed and approved the submitted version.

## Conflict of interest

The authors declare that the research was conducted in the absence of any commercial or financial relationships that could be construed as a potential conflict of interest.

## Publisher’s note

All claims expressed in this article are solely those of the authors and do not necessarily represent those of their affiliated organizations, or those of the publisher, the editors and the reviewers. Any product that may be evaluated in this article, or claim that may be made by its manufacturer, is not guaranteed or endorsed by the publisher.

## Abbreviations

BV, bevacizumab; CRC, colorectal cancer; EGFR, epidermal growth factor receptor; HR, hazard ratio; KRAS, kirsten rat sarcoma viral oncogene homologue; m, median; moAb, monoclonal antibody; MSI, microsatellite instability; OS, overall survival; PFS, progression-free survival; WT, wild-type; 95% CI, 95% confidence interval.
