# Supplementary figures and images for: Mutations matter: An observational study of the prognostic and predictive value of KRAS mutations in metastatic colorectal cancer
Source: Front Oncol. 2022 Nov 29;12:1055019. doi: 10.3389/fonc.2022.1055019 (PMC9745189; doi:10.3389/fonc.2022.1055019)

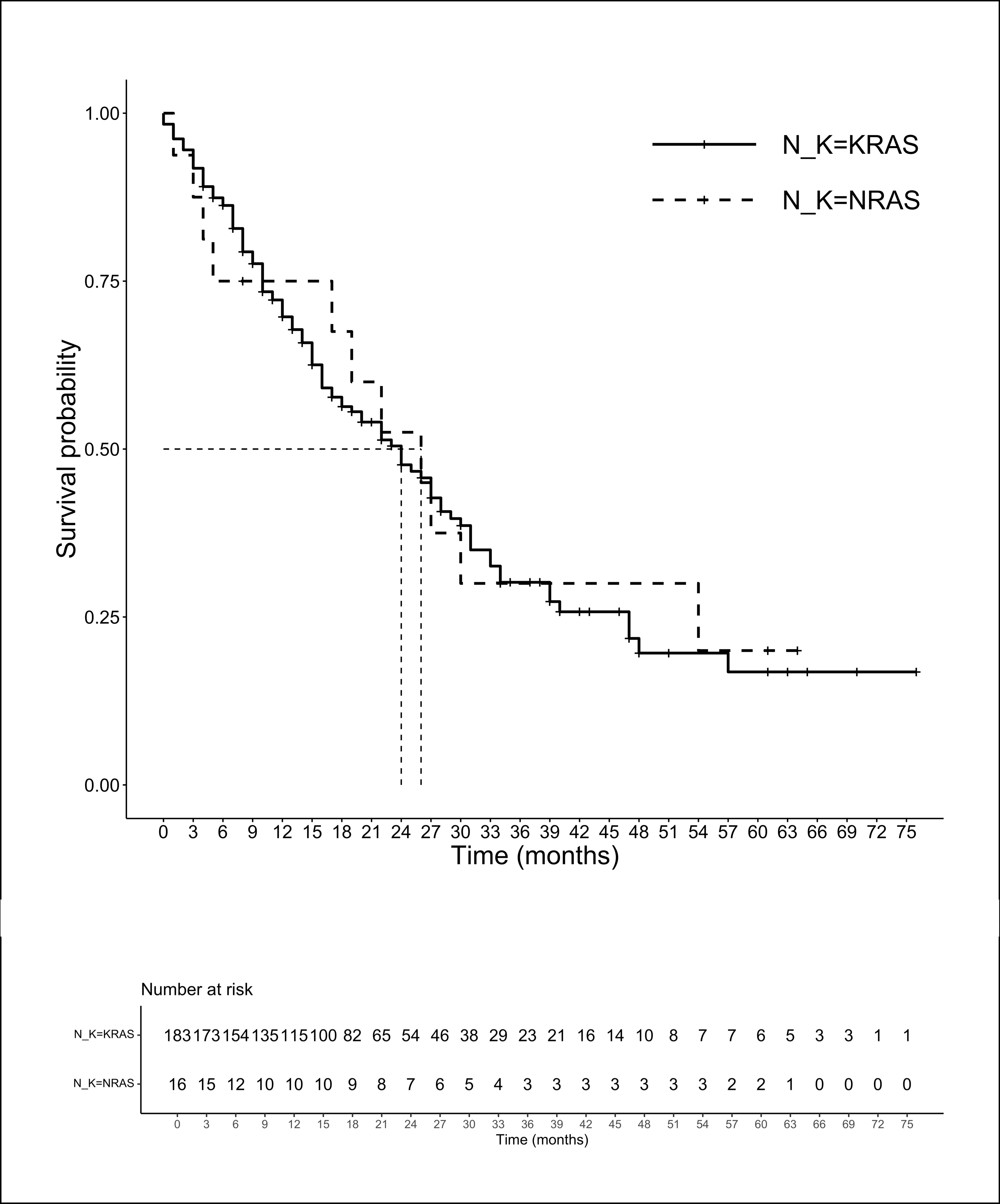

Supplement: Supplementary file 1 [file Image_1.jpeg]
